# Age–Stage, Two-Sex Life Table Analysis of *Riptortus pedestris* (Hemiptera: Alydidae) Across Different Soybean Varieties

**DOI:** 10.3390/insects15120952

**Published:** 2024-11-30

**Authors:** Juan Cui, Jingxu Yin, Xinyue Tian, Yu Gao, Shusen Shi, Wenbo Li

**Affiliations:** 1 College of Agriculture, Jilin Agriculture Science and Technology College, Jilin 132101, China; cuijuanjilin@163.com (J.C.); yinjingxu20040508@163.com (J.Y.); 2 Key Laboratory of Soybean Disease and Pest Control, Ministry of Agriculture and Rural Affairs, Changchun 130118, China; gaoy1101@163.com (Y.G.); sss-63@263.net (S.S.); 3 School of Grassland Science, Beijing Forestry University, Beijing 100091, China; txy18943655384@163.com; 4 College of Plant Protection, Jilin Agricultural University, Changchun 13118, China; 5 College of Biological Resource and Food Engineering, Qujing Normal University, Qujing 655011, China

**Keywords:** *Riptortus pedestris*, developmental period, population adaptability, fecundity, life table

## Abstract

*Riptortus pedestris* (Hemiptera: Alydidae) is an economically important pest of soybeans, significantly affecting their quality and market value. This study aims to evaluate the adaptability of *R. pedestris* to different soybean varieties and provide a foundation for selecting resistant varieties and developing effective integrated pest management strategies. Seven widely planted varieties (Beidou No. 3, Jiyu 47, Jiyu No. 80, Jiyu No. 90, Jiyu 203, Kangxianchong No. 6, and Kangxianchong 12) were selected for testing, and the developmental and population parameters of *R. pedestris* were measured using an age–stage, two-sex life table method. Notable differences were found in the developmental period, female longevity, and total preoviposition period (TPOP) of *R. pedestris* across diverse varieties. For those feeding on Kangxianchong 12, the nymph stage (14.73 ± 0.11 days), total longevity (37.35 ± 1.12 days), total female longevity (38.83 ± 1.80 days), and fecundity (38.86 ± 3.27 eggs per female) were all remarkably longer than on the other varieties. Additionally, the intrinsic rate of increase (*r*), finite rate of increase (*λ*), net reproductive rate (*R*_0_), and average generation time (*T*) of *R. pedestris* varied significantly between soybean varieties. The lowest *r* (0.0789 ± 0.0075 per day) and *λ* (1.0821 ± 0.0081 per day) values were recorded for Kangxianchong No. 6, while the highest were for Jiyu 47 (*r* = 0.0933 ± 0.0070 per day, *λ* = 1.0978 ± 0.0076 per day). The *R*_0_ and *T* values for *R. pedestris* on Kangxianchong 12 (*R*_0_ = 16.33 ± 3.24 offspring per female, *T* = 31.18 ± 0.79 days) were remarkably higher than those of the other six varieties, suggesting that Kangxianchong 12 and Jiyu 47 are preferred hosts, whereas Kangxianchong No. 6 is less preferred. These results provide valuable insights for optimizing soybean cultivation and managing *R. pedestris*.

## Simple Summary

*Riptortus pedestris* (Hemiptera: Alydidae) is a significant pest of soybeans. In this study, seven soybean varieties were selected to assess the adaptability of *R. pedestris* to different varieties, with biological parameters measured using an age–stage, two-sex life table method. Among the seven varieties, Kangxianchong 12 exhibited significantly longer durations for the nymph stage, total longevity, female longevity, and fecundity compared to the other six varieties. Jiyu 47 had the highest values for the intrinsic rate of increase (*r*) and the finite rate of increase (*λ*), while Kangxianchong 12 had markedly higher net reproductive rate (*R*_0_) and mean generation time (*T*) values. These findings suggest that Kangxianchong 12 and Jiyu 47 are the most suitable hosts for *R. pedestris*. Our study provides a foundation for screening soybean varieties for resistance and developing integrated pest management strategies. Planting varieties with low adaptability to *R. pedestris* could be an effective approach to reducing its population in soybean-growing regions.

## 1. Introduction

The bean bug, *Riptortus pedestris* (Hemiptera: Alydidae), is a major pest of soybeans, widely distributed across East and Southeast Asia, including India, Japan, South Korea, Pakistan, Sri Lanka, Myanmar, Malaysia, and China [1,2,3,4]. It has been recorded on more than 30 plant species from 13 families, primarily legumes, with soybeans being one of its most suitable hosts [4,5]. In China alone, *R. pedestris* has been reported to cause damage in over 20 provinces [4]. Both nymphs and adults feed by inserting their sucking mouthparts into seeds, pods, stems, and leaves, extracting nutrients and water, which results in significant yield losses and reduced seed quality [6].

Soybean pods at the pod stage can fully satisfy the nutritional requirements for the development and reproduction of *R. pedestris* at each life stage. During the podding stage, the pods serve as a primary source of nutrition for the pest population in the field [7]. Field cage tests have shown that the reproductive growth stage (R4 stage), when seeds are developing and nutrients are accumulating, is particularly susceptible to damage from *R. pedestris* [8]. This aligns with field observations of *R. pedestris* population dynamics, where adults and eggs are most commonly found between the R2–R4 stages of soybean growth, peaking in the R5 stage, while nymphs start to appear in the R6 stage [9]. Direct feeding by *R. pedestris* is the main cause of the “staygreen syndrome” in soybeans, with the most critical feeding damage occurring during the early pod stage [10]. In 2010, over 50% of soybean fields suffered varying levels of damage from *R. pedestris*, and staygreen syndrome was reported in a wide range of soybean cultivars [11]. In severe outbreak conditions, the incidence of staygreen syndrome can reach as high as 90%, leading to near-complete yield loss [12,13].

Developing crop resistance through breeding has long been acknowledged as one of the most cost-efficient and environmentally sustainable methods for managing insect pest populations, making it a key component of sustainable integrated pest management (IPM) programs [14,15]. Plant characteristics that provide resistance to herbivorous insects include physical structures, such as trichomes, waxy coatings, and thickened leaf margins, as well as biochemical compounds that can be toxic or repellent to herbivores [16]. Moreover, plants can activate indirect defenses by emitting volatile compounds that draw in natural enemies, including predators and parasitoids, which attack the herbivores [17]. Plant resistance can impact arthropod herbivores by reducing their preference for the plant, reducing their fitness on resistant plants, or minimizing the fitness costs of herbivore-triggered damage to the plant [18,19,20,21].

There are substantial differences in the nutritional composition of various soybean varieties [22], which creates favorable conditions for selecting insect-resistant varieties. These resistant varieties can serve as an effective tool for controlling insect pests like the bean bug. For example, Wada et al. found that the small-seeded soybean cultivar Kyushu-143, when planted at the normal sowing time, showed less seed damage from soybean bugs compared to the larger-seeded cultivars, including Sachiyutaka and Fukuyutaka [23]. Although the nymphal development time and fecundity of *R. pedestris* were not significantly influenced by various soybean varieties, nymphal mortality and adult female size showed notable differences [9]. Resistance traits such as hilum characteristics, pod length, and pubescence color have been found to correlate with cultivar resistance to *R. pedestris* [24]. Soybean varieties with denser and longer pod hairs, as well as thicker pod skins, generally exhibited milder damage, demonstrating stronger resistance [25]. However, despite these findings, the mechanisms underlying *R. pedestris* adaptation to different host plants remain unclear.

A life table is a crucial tool for assessing the relative suitability of host plants for pests [26]. However, traditional life tables, which overlook male populations, varying developmental stages, and individual differences, have been shown to inaccurately describe insect population dynamics [27]. The age–stage, two-sex life table is commonly recommended, as it accounts for both sexes and eliminates many of these inherent limitations [28]. This method has been used to study a variety of insect pests, including *Spodoptera frugiperda* reared on different plant cultivars [29,30], *Plutella xylostella* fitness on primary and marginal hosts [31], host-switching in *Cnaphalocrocis medinalis* [32], and the effect of temperature on *Halyomorpha halys* [33]. It has also been successfully applied to *R. pedestris*, including studies on population fitness under fluctuating and constant temperatures [34,35] and the impact of pesticide concentrations on population dynamics [36]. However, no age–stage, two-sex life table analysis has yet been conducted for *R. pedestris* on different soybean varieties.

This research was conducted with the hypothesis that different soybean varieties influence the development of *R. pedestris*. To test this, the effects of various soybean varieties on the biological characteristics of *R. pedestris* were assessed using a free-choice test and the age–stage, two-sex life table method. The findings offer new insights for optimizing soybean cultivation and serve as a foundation for effective management and control strategies for *R. pedestris*.

## 2. Materials and Methods

### 2.1. Insect Population Rearing

Adult *R. pedestris* specimens were harvested from a soybean field at Nanjing (32°2′14″ N, 118°50′4″ E), Jiangsu Province, China, in 2022. The adults were transferred into nylon mesh cages (50 cm × 50 cm × 50 cm) and then exposed to moistened soybean seeds that had been immersed in water for 48 h, following the method described by Tian et al. (2020) [35]. Additionally, potted soybean plants (var. Jinong 38) with pods were placed in the cages. The moistened seeds and plants were replaced weekly. When the adults laid eggs, the plants were transferred to a different cage as necessary. Colonies were maintained at 25 ± 1 °C, 60 ± 5% humidity, and 16:8 h light/dark. The insects were reared for three consecutive generations for use in the experiments. All experiments and rearing procedures were conducted at Jilin Agricultural University, Changchun, China.

### 2.2. Host Plants

Seven soybean varieties (Beidou No. 3, Jiyu 47, Jiyu No. 80, Jiyu No. 90, Jiyu 203, Kangxianchong No. 6, and Kangxianchong 12) were obtained from the Key Laboratory of Soybean Disease and Pest Control at Jilin Agricultural University, Changchun, China. These varieties were selected based on their high grain yield and previous research findings. Seeds from the seven soybean varieties were sown in pots (28 cm diameter, 20 cm depth) for oviposition. The soybean hosts used for assessing the population development and growth of *R. pedestris* were cultivated at the experimental field of Jilin Agricultural University (43°48′47″ N, 125°25′4″ E).

### 2.3. Effects of Soybean Varieties on the Development and Population Parameters of R. pedestris

Seven soybean varieties were separately grown in 12-inch clay pots in a greenhouse at Jilin Agricultural University. Once the plants reached approximately 30 cm in height, they were transferred to separate insect-rearing cages (50 cm × 50 cm × 50 cm). Ten healthy pairs of *R. pedestris* were released on each plant for oviposition. For a life table study, we collected one hundred recently deposited eggs from each soybean variety within 12 h of successful oviposition. The collected eggs were placed in individual Petri dishes (diameter 9 cm) lined with moistened filter paper under controlled conditions of 25 ± 2 °C, 60 ± 5% RH, 16 h:8 h (L:D) photoperiod. The incubation duration and hatchability of the eggs were measured regularly every twelve hours. After hatching, sixty healthy nymphs were selected per treatment and deposited in separate 10 mL conical-bottomed centrifuge tubes, with one nymph per tube. Throughout the experiment, fresh soybean pods (3–4 cm, R5 growth stage) from the seven tested soybean varieties were provided as food and substituted every 2–3 days. Developmental time of nymphs and survival were recorded daily by monitoring the presence of shed exuviae and observing nymphal characteristics. The presence of shed exuviae marked the successful completion of an instar, and the exuviae were promptly removed. Each newly emerged adult was collected every 24 h, sexed, and weighed on an electronic balance (Sartorius AL-204). The emerging adults from the same soybean variety were mated and kept separately in a translucent plastic container (14 cm tall, 9 cm top diameter, 10 cm base diameter) and fed fresh soybean pods. The cumulative number of eggs laid by each female daily; the duration of the preoviposition, oviposition, and postoviposition periods; and the egg hatching rate were recorded until both the female and male adults died.

### 2.4. Life Table Data Analysis

The life table data for *R. pedestris* were assessed using the age–stage, two-sex life table method, as described in previous studies [29,37,38], via the TWOSEX-MSChart (Ver. 5/7/2024) [39]. Key parameters calculated included the age–stage-specific survival rate (*S_xj_*), age-specific survival rate (*l_x_*), female age-specific fecundity (*m_x_*), net reproductive rate (*R*_0_), intrinsic rate of increase (*r*), finite rate of increase (*λ*), and mean generation time (*T*), following the procedures outlined by Chi and Liu [38].

*S_xj_* represents the probability that a newly laid egg will survive to age *x* and stage *j* (where *x* and *j* are age in days and the developmental stage, respectively). *l_x_*, which measures the survival probability from age 0 to *x*, was determined using the following formula:

(1) $$l_{x}=\sum_{j=1}^{\beta }s_{xj}$$

*f_xj_* refers to the number of eggs laid per day by a female at *x* and *j*. *m_x_*, representing the average number of eggs an individual produces at age *x*, was determined as follows:

(2) $$M_{x}\;=\;\frac{\sum_{j=1}^{\beta }S_{xj}f_{xj}}{\sum_{j=1}^{\beta }S_{xj}}$$

The product of *l_x_* and *m_x_* represents the age-specific net maternity (*l_x_m_x_*). The total of *l_x_m_x_* across all ages provides *R*_0_, which reflects the overall number of offspring an individual is expected to generate over its lifetime, and was determined as follows:

(3) $$R_{0}=\sum_{x=0}^{\infty }l_{x}m_{x}$$

*r* was determined using the Lotka–Euler equation with age starting from 0 [40] as follows:

(4) $$\sum_{x=0}^{\infty }e^{-r(x+1)}l_{x}m_{x}=1$$

*λ* was determined as follows:

*λ* = *e^r^* (5)

*T* represents the average time required for a population to increase by a factor of *R*_0_, assuming the population has achieved a stable age–stage distribution. It was determined using the following formula:

(6) $$T=\frac{\ln(R_{0})}{r}$$

The age–stage-specific life expectancy (*e_xj_*) indicates the expected remaining lifespan of an individual at *x* and *j* [41]. It was calculated as follows:

(7) $$e_{xj}=\sum_{i=x}^{\infty }\sum_{j=y}^{\beta }{S^{\prime }}_{iy}$$

where *e_xj_* refers to the likelihood that an individual at *x* and *y* will survive to reach *i* and *j*, calculated under the assumption that ${S^{\prime }}_{iy}$ = 1. The age–stage reproductive value (*v_xj_*) represents the expected contribution of an individual at *x* and *y* to the future population, and is determined as follows [42]:

(8) $$\upsilon_{xj}=\frac{e^{r\left(x+1\right)}}{S_{xj}}\sum_{i=x}^{\infty }e^{-r\left(i+1\right)}\sum_{y=j}^{\beta }{S^{\prime }}_{iy}f_{iy}$$

### 2.5. Statistical Analysis

For each life stage, developmental duration, adult weight, and reproductive parameters (fecundity and oviposition) among insects fed on the seven soybean varieties were compared using one-way analysis of variance (ANOVA), followed by Tukey’s HSD test for pairwise mean comparisons at a 5% significance level. All statistical analyses were performed with SPSS software (v27.0).

Life table parameters other than the developmental time for *R. pedestris* were analyzed using the TWOSEX-MSChart program [39]. Given that bootstrap analysis involves random resampling, paired bootstrap tests (100,000 runs) were conducted to minimize error values and assess variances and standard errors [18,19]. Figures and graphs were generated with SigmaPlot v14.0, which was also used to construct all diagrams.

## 3. Results

### 3.1. Influence of Soybean Varieties on the Developmental Duration of R. pedestris

The impact of different soybean varieties on the developmental time of *R. pedestris* is summarized in Table 1. All tested varieties supported the complete development of *R. pedestris* from egg to adult, though the specific diet of both nymphs and adults significantly influenced several developmental parameters. Developmental time varied significantly among the seven soybean varieties (Table 1). As expected, soybean variety had a notable effect on the developmental time of the first instar (*p* = 0.042), second instar (*p* < 0.001), third instar (*p* < 0.001), fourth instar (*p* < 0.001), and fifth instar (*p* < 0.001). A significant difference was also observed in the overall nymphal developmental time (*p* < 0.001). The longest nymphal developmental period was recorded on Kangxianchong 12 (14.73 ± 0.11 days), while the shortest was on Jiyu No. 80 (12.65 ± 0.12 days). The total longevity on Kangxianchong 12 (37.35 ± 1.12 days) was markedly longer than on the other varieties (*p* < 0.001), although no obvious differences were found in the total longevity among the remaining six varieties.

### 3.2. Biological Parameters of R. pedestris Fed on Various Soybean Varieties

Female adult longevity was highest on the Kangxianchong 12 variety (Table 2), with a statistically significant difference compared to the other six varieties (*p* < 0.001). The total female and male adult longevities on Kangxianchong 12 were 38.83 ± 1.80 days and 35.88 ± 1.30 days, respectively. No obvious differences were found in the total male longevity across the seven varieties. Interestingly, female longevity on Kangxianchong 12 exceeded that of males, whereas the opposite was observed for Kangxianchong No. 6. The remaining five varieties exhibited similar male and female longevity patterns.

The preoviposition period (APOP) of *R. pedestris* was shortest on Jiyu 47 (3.70 ± 0.17 days) and longest on Beidou No. 3 (4.19 ± 0.25 days), though no obvious differences were found among the seven varieties (*p* > 0.05). The total preoviposition period (TPOP) was longest on Kangxianchong 12 (25.71 ± 0.36 days), followed by Jiyu No. 90 and Kangxianchong No. 6, with no significant differences between them. Fecundity was highest on Kangxianchong 12 (38.86 ± 3.27 eggs per female), significantly higher (*p* = 0.008) than on Jiyu No. 80, Jiyu No. 90, Jiyu 203, and Kangxianchong No. 6.

Eggs hatched successfully on all seven soybean varieties. The highest hatching rate was recorded on Jiyu No. 90 (81.19 ± 1.53%), followed by Jiyu 47 (74.90 ± 3.33%) and Beidou No. 3 (70.31 ± 1.83%). The hatching rate on Jiyu No. 90 was significantly higher than on Jiyu No. 80, Jiyu 203, Kangxianchong No. 6, and Kangxianchong 12 (*p* < 0.001). The lowest hatching rate was observed on Kangxianchong No. 6 (47.46 ± 1.96%).

### 3.3. Weight of Adult R. pedestris Reared on Different Soybean Varieties

**Figure 1:**
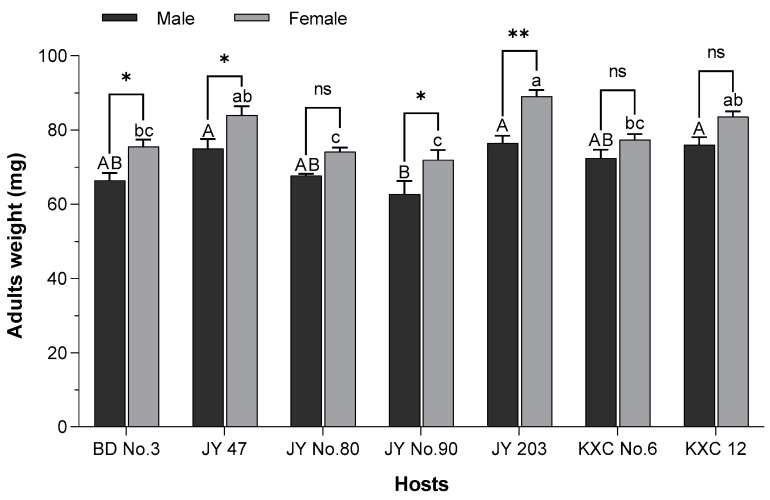
The male and female weights of *R. pedestris* fed on seven different soybean varieties. BD No.3: Beidou No. 3, JY 47: Jiyu 47, JY No. 80: Jiyu No. 80, JY No.90: Jiyu No. 90, JY 203: Jiyu 203, KXC No.6: Kangxianchong No. 6, KXC 12: Kangxianchong 12. The data are presented as mean ± SE. Significant differences in female weight among the different soybean varieties are indicated by different capital letters, according to Tukey’s test (*p* < 0.05). For male weight, significant differences among soybean varieties are marked by different lowercase letters at the 0.05 significance level (*p* < 0.05). * *p* < 0.05, ** *p* < 0.001, ns, not significant, between males and females fed on the same soybean varieties.

The adult weights of insects reared on various soybean varieties are shown in Figure 1. Across all seven soybean varieties, female insects were heavier than males. Significant differences in adult weight were observed for both male and female insects across the different soybean varieties. Specifically, females and males reared on Jiyu 203 and Kangxianchong 12 were significantly heavier than those reared on Jiyu No. 90 (*p* < 0.001). Female adult weights were significantly higher than male adult weights on Beidou No. 3 (*p* < 0.005), Jiyu 47 (*p* < 0.005), Jiyu No. 90 (*p* < 0.005), and Jiyu 203 (*p* < 0.001). These differences in adult weights may be attributed to variations in the nutritional content of the different soybean varieties.

### 3.4. Life Table and Population Parameters of R. pedestris Reared on Various Soybean Varieties

The population parameters of *R. pedestris* are presented in Table 3. The *R*_0_ and *T* were significantly higher on Kangxianchong 12 compared to other varieties. The highest *λ* and *r* were observed on Jiyu 47, except for Kangxianchong 12. The lowest values for *R*_0_ (9.67 ± 1.98 offspring per female), *r* (0.0789 ± 0.0075 per day), and *λ* (1.0821 ± 0.0081 per day) were recorded on Kangxianchong No. 6. Additionally, the shortest *T* (27.54 ± 0.28 days) was found in *R. pedestris* reared on Jiyu No. 80. Across all varieties, *r* and *λ* were consistently greater than 0 and 1, respectively, indicating that *R. pedestris* can survive on these varieties.

**Figure 2:**
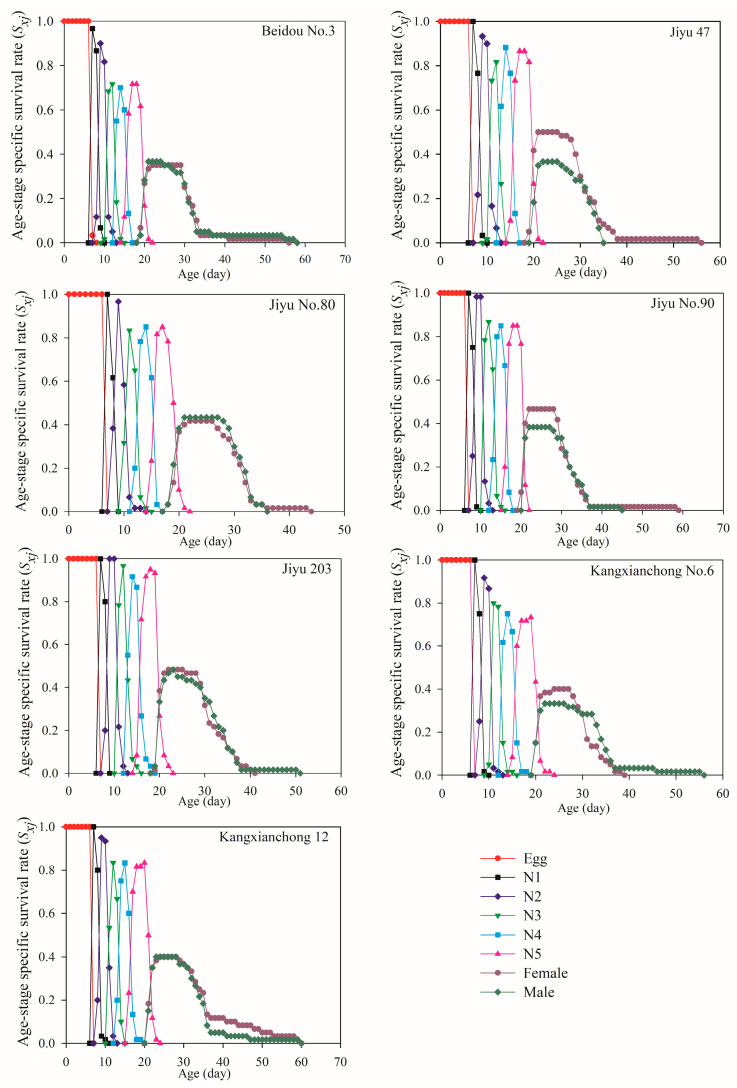
The *S_xj_* values of *R. pedestris* reared on seven soybean varieties. Note: N1–N5 denote the 1st, 2nd, 3rd, 4th, and 5th instars, respectively.

The values of *S_xj_* on the seven different soybean varieties are shown in Figure 2. Due to individual variations in developmental rates, overlaps occurred in the survival curves across stages. The highest nymph survival rate was observed on Jiyu 203, with 95% of the eggs developing into the adult stage, followed by Jiyu 47, whereas the lowest nymph survival rate was recorded on Beidou No. 3 (71.67%). *R*. *pedestris* reared on Jiyu 203 exhibited the highest adult survival rate, with the lowest recorded on Beidou No. 3. The overall survival duration was longer in adult males than in adult females for *R. pedestris* fed on Beidou No. 3, Jiyu 203, Kangxianchong No. 6, and Kangxianchong 12. Additionally, on each soybean variety, males and female adults appeared on the same day except Jiyu 47 and Jiyu No. 90, on which the female adults appeared 1 d earlier than males.

**Figure 3:**
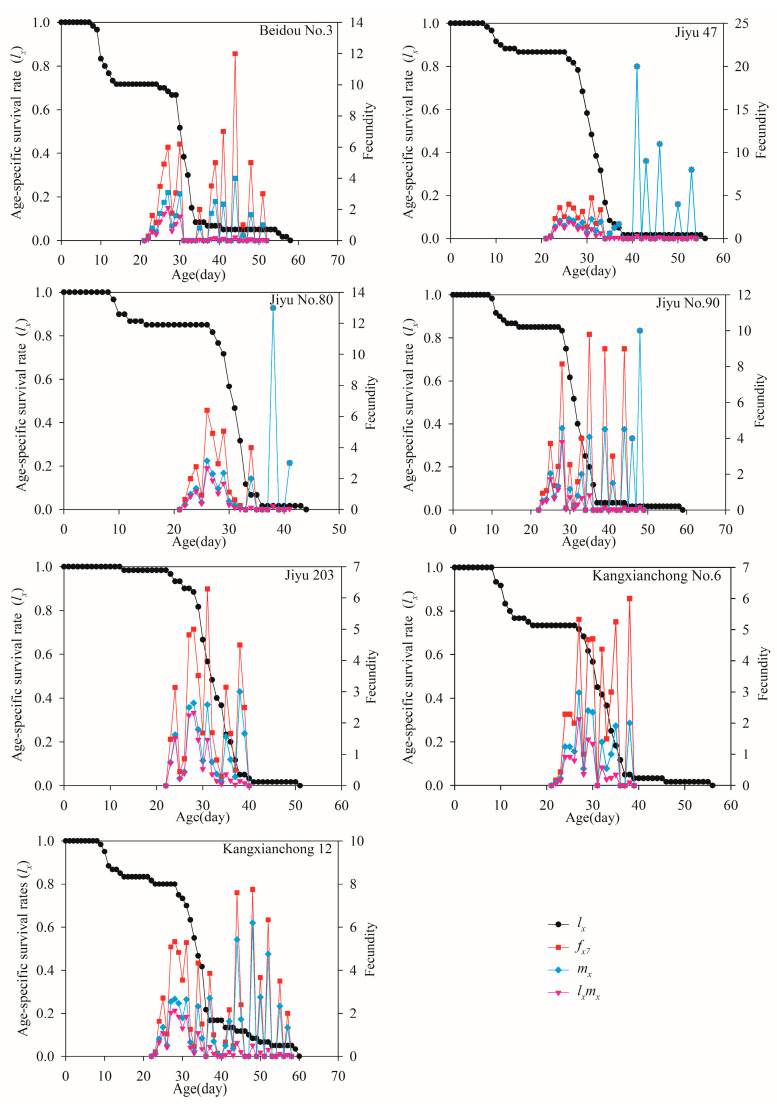
The *l_x_*, *f_x_*_7_, *m_x_*, and *l_x_m_x_* values of *R. pedestris* were reared on seven soybean varieties.

The *l_x_* values of the *R. pedestris* population gradually decreased with increasing *x* (Figure 3). On Jiyu 203, the *l_x_* curve showed only a slight decrease during the first 25 days, indicating a low mortality rate during this period. Following day 25, the *l_x_* curve continued to decline steadily, with a sharp drop observed on day 30 for Beidou No. 3, Jiyu 47, Jiyu No. 80, and Jiyu No. 90, suggesting a high mortality rate in the adult stage. The *f_x_*_7_ (females represent stage 7 in the life cycle and *x* denotes age) curve peaked on Jiyu 47, which was higher than on other host plants. The *m_x_* curve revealed that reproduction began at age 22 days on Beidou No. 3, Jiyu 47, Jiyu No. 80, and Kangxianchong No. 6, and at age 23 days on the other varieties. However, multiple peaks were found in the *m_x_* curve, indicating variations in the oviposition periods of individuals. As illustrated in Figure 3, the *l_x_m_x_* value reached its peak on day 26 for Jiyu 47 and Jiyu No. 80, on day 27 for Beidou No. 3 and Kangxianchong No. 6, and on day 28 for Jiyu No. 90, Jiyu 203, and Kangxianchong 12.

**Figure 4:**
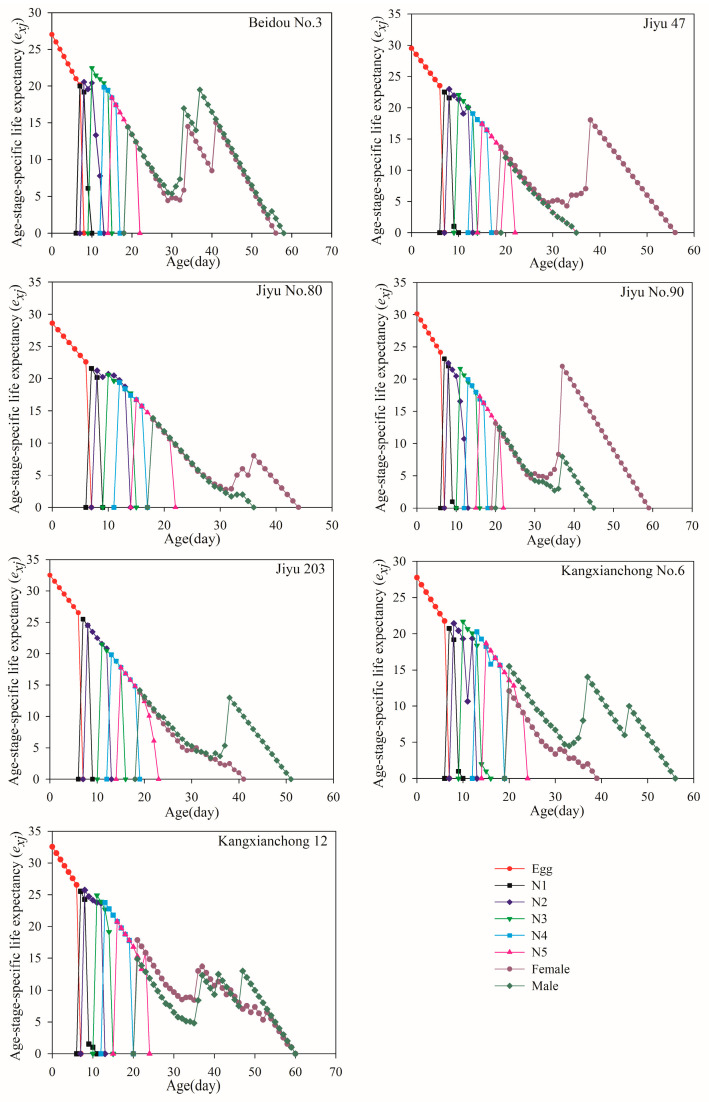
The *e_xj_* value of each age–stage group of *R. pedestris* reared on seven soybean varieties. Note: N1–N5 denote the 1st, 2nd, 3rd, 4th and 5th instar, respectively.

As shown in Figure 4, the *e_xj_* values for all individuals reared on soybean exhibited a downward trend, gradually decreasing to 0 with increasing age. The average life expectancy values of *R. pedestris* individuals that were fed on Beidou No. 3, Jiyu 47, Jiyu No. 80, Jiyu No. 90, Kangxianchong No. 6, and Kangxianchong 12 were 27.02, 29.50, 28.60, 30.15, 32.50, 27.73, and 32.53 d, respectively. Among adults, the highest *e_xj_* was observed in male adults on Kangxianchong No. 6 (15.50 days) and in female adults on Kangxianchong 12 (17.83 days). The lowest *e_xj_* was recorded in male adults on Jiyu 47 (12.00 days) and female adults on Jiyu No. 90 (13.14 days). Across all seven soybean varieties, female adults generally had a higher *e_xj_* than males, except on Jiyu 47, Jiyu No. 90, and Kangxianchong 12.

**Figure 5:**
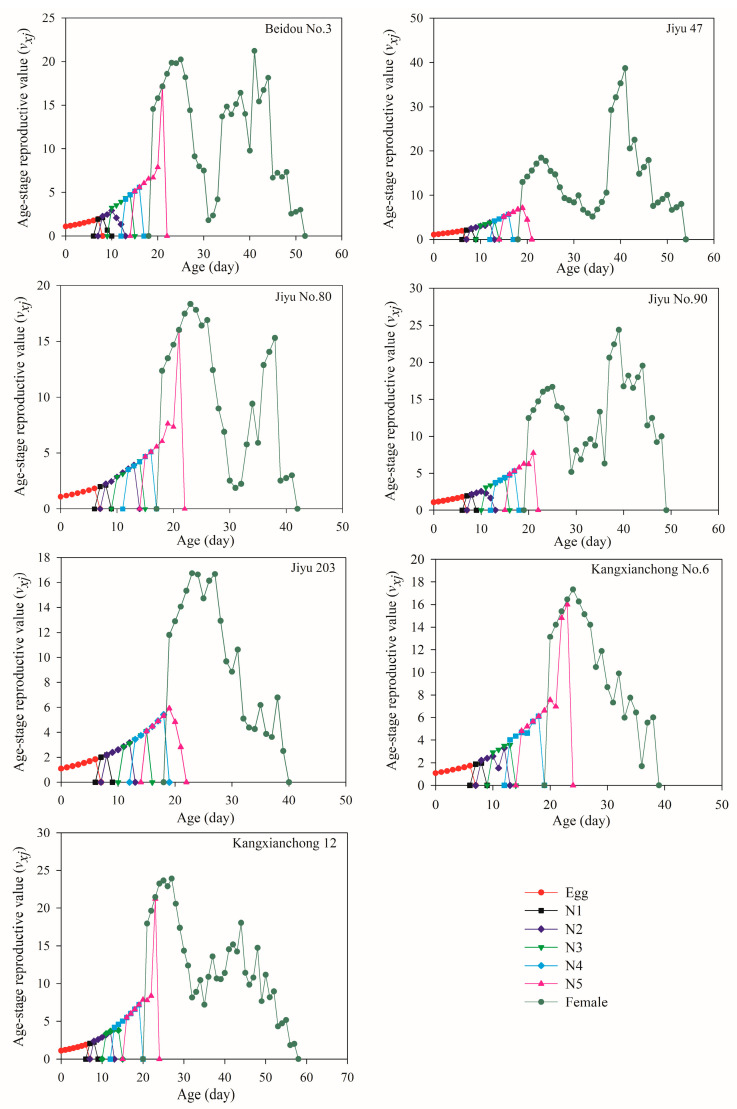
The *v_xj_* value of each age–stage group of *R. pedestris* reared on seven soybean varieties. Note: N1–N5 denote the 1st, 2nd, 3rd, 4th, and 5th instar, respectively.

The *v_xj_* values of *R. pedestris* initially increased and then declined to 0 as the development stage progressed (Figure 5). The highest reproductive peak for female adults occurred at 21 days on Beidou No. 3 (20.25), 41 days on Jiyu 47 (38.70), 24 days on Jiyu No. 80 (17.82), 39 days on Jiyu No. 90 (24.39), 23 days on Jiyu 203 (16.73), 24 days on Kangxianchong No. 6 (17.33), and 27 days on Kangxianchong 12 (23.90). Among the varieties, the highest *v_xj_* was observed on Jiyu 47, while the lowest was recorded on Jiyu 203.

## 4. Discussion

Insect–plant interactions play a critical role in developing sustainable pest control strategies [43]. This challenge has driven researchers to seek effective alternative control methods, such as enhancing host plant resistance [44,45,46,47,48]. For example, in Southeast Asian countries, current rice production largely depends on the use of resistant rice varieties to manage rice planthopper populations [49]. In Northeast China, there is a strong emphasis on promoting soybean varieties resistant to the soybean pod borer [50]. Li Feng et al. reported significant differences in *R. pedestris* responses across soybean varieties, with Jidou 17 showing higher susceptibility compared to Zhonghuang 39 [51]. Wada et al. found that the Japanese small-grained cultivar (Kyushu-143) was less affected by pest pressures than the large-grained cultivars (Fukuyutaka and Sachiyutaka) [23]. Previous research has shown that host plant suitability is critical during early larval stages, as feeding on unsuitable hosts can adversely affect insect life history traits [52,53,54]. Additionally, Park et al. observed that the development time and survival rate of *R. pedestris* was low in Daepung-2ho compared to the other six cultivars of soybeans, and in the field, the least damaged soybean cultivar was Daepung-2ho [1]. The results of this experiment showed significant differences in the growth and reproductive parameters of *R. pedestris* when fed on seven different soybean varieties. Specifically, growth, development, and fecundity varied among varieties. For instance, the number of eggs produced by a single female was significantly higher on Kangxianchong 12 compared to most other varieties, with the exception of Jiyu 47 and Beidou No. 3. These findings indicate that different soybean varieties can affect the growth, development, and reproduction of *R. pedestris*.

In this study, *R. pedestris* fed on Kangxianchong 12 exhibited the highest adult weight, longest nymphal stage duration, greatest adult longevity, and maximum egg production per female. The extended larval development on a high-quality host plant may act as a compensatory mechanism, allowing the larvae to gain additional weight and successfully complete their life cycle [55]. *R. pedestris* adults fed on Kangxianchong 12 had higher weights and fecundity than those on the other six soybean varieties, suggesting a positive relationship between these two parameters. Similarly, Kim et al. observed that female adults raised on the Agakong variety were larger and that *R. pedestris* showed a preference for the seeds of the Cheongjakong variety [9]. This suggests that *R. pedestris* exhibits selective preference for certain soybean varieties. In this study, adults fed on Kangxianchong No. 6 had the lowest egg production and hatching rates. Interestingly, although *R. pedestris* laid the fewest eggs on Jiyu 90, the hatchability of the next generation was highest, suggesting that specific compounds in this variety might be critical for egg development.

The life table parameters are key indicators that describe the growth potential of a population, reflecting the combined effects of population growth, survival, and reproductive capacity on overall population fitness [21,55]. Our study demonstrated that *R. pedestris* populations were able to complete their life cycle on each of the seven soybean varieties; however, significant differences were observed in survival rate, developmental period, reproduction, and population growth depending on the host plant. The *R*_0_, *r*, and *λ* were lowest for *R. pedestris* on Kangxianchong No. 6. The highest *R*_0_ was recorded on Kangxianchong 12, followed by Jiyu 47. Generally, the intrinsic growth rate of insect populations reflects both their adaptability to a host and feeding preference. Thus, our findings suggest that *R. pedestris* displayed strong adaptability to Jiyu 47 and Kangxianchong 12, while Kangxianchong No. 6 exhibited the highest resistance to *R. pedestris*.

The *S_xj_* curves displayed overlapping trends, reflecting variations in developmental rates among *R. pedestris* individuals, similar to findings reported in prior studies [56]. The highest survival rate for *R. pedestris* was observed on Jiyu 203 (96.67%), followed by Jiyu 47 (92.86%). Both *e_xj_* and *v_xj_* values varied among individuals at the same age but different developmental stages, consistent with previous observations [57]. The *e_xj_* values showed a decreasing trend across all soybean varieties, with *R. pedestris* on Kangxianchong 12 exhibiting the longest average *e_xj_* (32.53 days), while the shortest was observed on Beidou No. 3 (27.02 days). The *v_xj_* values indicated significant troughs on Beidou No. 3 and Jiyu No. 80, indicating multiple peaks on these varieties. The *e_xj_* was assessed using the *S_xj_*, assuming a constant age–stage distribution within the population, which could be useful for predicting population survival under those conditions.

## 5. Conclusions

The life cycle of *R. pedestris* populations was completed on all soybean varieties; however, variations were observed in survival rate, development, reproduction, and population growth among the seven soybean varieties. When reared on Jiyu 47, *R. pedestris* exhibited a shorter developmental period, higher reproductive capacity, greater preadult survival, higher hatching rates in the next generation, and increased population growth parameters (*r*, *λ*). On Kangxianchong 12, *R. pedestris* had a longer developmental period, the highest reproductive capacity, higher preadult survival, and the highest population growth parameters (*r*, *λ*, *R*_0_, *T*). In contrast, *R. pedestris* reared on Kangxianchong No. 6 had the shortest developmental period, the lowest reproductive capacity, lower preadult survival, the lowest hatching rate, and the lowest population growth parameters (*R*_0_, *r*, *λ*). Based on these results, Kangxianchong 12 is the most favorable host for *R. pedestris*, followed by Jiyu 47, while Kangxianchong No. 6 is the least suitable. These findings provide important insights for predicting *R. pedestris* population dynamics and are valuable for developing integrated pest management strategies targeting this pest across different soybean varieties.

## Figures and Tables

**Table 1:** Mean developmental time and longevity of *R. pedestris* reared on seven soybean varieties.

Developmental Stage (Days)	*n*	Beidou No. 3	*n*	Jiyu 47	*n*	Jiyu No. 80	*n*	Jiyu No. 90	*n*	Jiyu 203	*n*	Kangxianchong No. 6	*n*	Kangxianchong 12
Egg	60	7.00 ± 0.00 ^a^	60	7.00 ± 0.00 ^a^	60	7.00 ± 0.00 ^a^	60	7.00 ± 0.00 ^a^	60	7.00 ± 0.00 ^a^	60	7.00 ± 0.00 ^a^	60	7.00 ± 0.00 ^a^
1st instar	55	1.87 ± 0.05 ^a^	56	1.77 ± 0.06 ^ab^	58	1.60 ± 0.06 ^b^	59	1.75 ± 0.06 ^ab^	60	1.80 ± 0.05 ^ab^	55	1.75 ± 0.06 ^ab^	57	1.79 ± 0.05 ^ab^
2nd instar	45	2.20 ± 0.07 ^bc^	53	2.45 ± 0.10 ^ab^	54	2.11 ± 0.08 ^c^	53	2.36 ± 0.07 ^abc^	60	2.45 ± 0.07 ^ab^	49	2.24 ± 0.07 ^abc^	52	2.65 ± 0.09 ^a^
3rd instar	44	2.18 ± 0.06 ^c^	53	2.08 ± 0.08 ^c^	52	2.10 ± 0.06 ^c^	52	2.69 ± 0.07 ^a^	59	2.31 ± 0.07 ^bc^	45	2.22 ± 0.06 ^bc^	50	2.46 ± 0.08 ^ab^
4th instar	43	2.74 ± 0.08 ^c^	52	2.73 ± 0.07 ^c^	51	2.86 ± 0.08 ^ab^	51	3.04 ± 0.05 ^a^	59	2.75 ± 0.07 ^ab^	44	2.96 ± 0.10 ^ab^	50	3.06 ± 0.07 ^a^
5th instar	43	4.09 ± 0.06 ^bc^	52	4.23 ± 0.07 ^b^	51	3.88 ± 0.10 ^c^	51	4.18 ± 0.06 ^bc^	58	4.05 ± 0.06 ^bc^	44	4.61 ± 0.09 ^a^	48	4.90 ± 0.07 ^a^
Nymph	43	13.09 ± 0.01 ^c^	52	13.27 ± 0.08 ^c^	51	12.65 ± 0.12 ^d^	51	14.04 ± 0.07 ^b^	58	13.29 ± 0.10 ^c^	44	13.73 ± 0.12 ^b^	48	14.73 ± 0.11 ^a^
Total longevity	43	33.44 ± 1.04 ^b^	52	32.40 ± 0.61 ^b^	51	31.75 ± 0.39 ^b^	51	33.31 ± 0.68 ^b^	58	33.00 ± 0.58 ^b^	44	33.64 ± 0.74 ^b^	48	37.35 ± 1.12 ^a^

Note: The data are mean ± SE, and significant differences at the 0.05 level are marked by different lowercase letters in the same row.

**Table 2:** The longevity and reproduction of *R. pedestris* reared on seven soybean varieties.

Developmental Stage	*n*	Beidou No. 3	*n*	Jiyu 47	*n*	Jiyu No. 80	*n*	Jiyu No. 90	*n*	Jiyu 203	*n*	Kangxianchong No. 6	*n*	Kangxianchong 12
Female adult longevity (d)	21	12.86 ± 0.62 ^b^	30	12.63 ± 0.97 ^b^	25	11.88 ± 0.69 ^b^	28	12.18 ± 0.51 ^b^	29	12.62 ± 0.70 ^b^	24	11.29 ± 0.64 ^b^	24	17.13 ± 1.18 ^a^
Male adult longevity (d)	21	13.32 ± 0.69 ^ab^	22	11.45 ± 0.43 ^b^	26	12.31 ± 0.45 ^ab^	23	12.39 ± 0.78 ^ab^	29	12.79 ± 0.65 ^ab^	20	14.85 ± 0.75 ^a^	24	14.13 ± 0.78 ^ab^
Female total longevity (d)	21	33.43 ± 1.25 ^b^	30	32.70 ± 0.97 ^b^	25	31.64 ± 0.68 ^b^	28	33.14 ± 1.07 ^b^	29	32.83 ± 0.69 ^b^	24	32.08 ± 0.62 ^b^	24	38.83 ± 1.80 ^a^
Male total longevity (d)	22	33.45 ± 1.67 ^a^	22	32.00 ± 0.57 ^a^	26	31.85 ± 0.40 ^a^	23	33.52 ± 0.76 ^a^	29	33.17 ± 0.95 ^a^	20	35.50 ± 1.37 ^a^	24	35.88 ± 1.30 ^a^
APOP (d)	21	4.19 ± 0.25 ^a^	30	3.70 ± 0.17 ^a^	25	3.80 ± 0.12 ^a^	28	3.93 ± 0.19 ^a^	29	4.07 ± 0.19 ^a^	24	3.92 ± 0.22 ^a^	24	4.04 ± 0.35 ^a^
TPOP (d)	21	24.29 ± 0.32 ^bc^	30	23.77 ± 0.20 ^c^	25	23.56 ± 0.18 ^c^	28	24.93 ± 0.25 ^ab^	29	24.28 ± 0.24 ^bc^	24	24.67 ± 0.28 ^abc^	24	25.71 ± 0.36 ^a^
Fecundity (eggs/♀)	21	29.11 ± 3.00 ^ab^	30	26.26 ± 3.70 ^ab^	25	24.17 ± 2.70 ^b^	28	23.73 ± 2.81 ^b^	29	25.15 ± 2.67 ^b^	24	23.64 ± 2.90 ^b^	24	38.86 ± 3.27 ^a^
Hatching rate (%)	147	70.31 ± 1.83 ^bc^	131	74.90 ± 3.33 ^ab^	145	62.65 ± 2.79 ^cd^	208	81.19 ± 1.53 ^a^	191	56.50 ± 2.21 ^de^	206	47.46 ± 1.96 ^e^	200	50.98 ± 2.24 ^e^

Note: The data are mean ± SE, and significant differences at the 0.05 level are marked by different lowercase letters in the same row.

**Table 3:** Population parameters of *R. pedestris* reared on various soybean varieties.

Hosts	Net Reproductive Rate (*R*_0_)	Mean Generation Time *T* (d)	Intrinsic Rate of Increase *r* (Per Day)	Finite Rate of Increase *λ* (Per Day)
Beidou No. 3	10.15 ± 2.05 ^c^	28.45 ± 0.63 ^bc^	0.0815 ± 0.0073 ^bc^	1.0849 ± 0.0079 ^bc^
Jiyu 47	13.97 ± 2.91 ^b^	28.25 ± 0.62 ^bc^	0.0933 ± 0.0070 ^a^	1.0978 ± 0.0076 ^a^
Jiyu No. 80	10.77 ± 2.08 ^c^	27.54 ± 0.28 ^c^	0.0863 ± 0.0071 ^b^	1.0901 ± 0.0077 ^b^
Jiyu No. 90	11.45 ± 2.15 ^c^	29.10 ± 0.64 ^b^	0.0838 ± 0.0066 ^bc^	1.0874 ± 0.0072 ^bc^
Jiyu 203	12.27 ± 2.12 ^c^	28.78 ± 0.28 ^b^	0.0871 ± 0.0061 ^b^	1.0910 ± 0.0067 ^b^
Kangxianchong No. 6	9.67 ± 1.98 ^c^	28.75 ± 0.36 ^b^	0.0789 ± 0.0075 ^c^	1.0821 ± 0.0081 ^c^
Kangxianchong 12	16.33 ± 3.24 ^a^	31.18 ± 0.79 ^a^	0.0896 ± 0.0065 ^a^	1.0937 ± 0.0071 ^a^

Note: The data are mean ± SE, and significant differences at the 0.05 level are marked by different lowercase letters in the same column.

## Data Availability

The data presented in this study are available on request from the corresponding author.
